# Significant Geographic Variation in the Epidemiology and Management of Pediatric Osteochondritis Dissecans Exists Between the Asia‐Pacific and North America

**DOI:** 10.1002/ars2.70096

**Published:** 2026-10-30

**Authors:** Joshua Rui Yen Wong, Yun Xiu Lam, Si Heng Sharon Tan, James Hoi Po Hui, Ming Wang

**Affiliations:** ^1^ Department of Orthopaedic Surgery University Orthopaedic, Hand and Reconstructive Microsurgery Cluster, National University Health System (NUHS) Singapore Singapore

## Abstract

**Purpose:**

To evaluate regional variations in epidemiology, diagnostic approaches, and surgical interventions for pediatric osteochondritis dissecans (OCD) between Asia‐Pacific (APAC) and North America (NA).

**Methods:**

A comprehensive search of PubMed, Scopus, and Cochrane Library databases was conducted from database inception until August 2025. Studies on epidemiology and surgical treatment of pediatric OCD were screened independently by 2 reviewers. Data was extracted and collated according to Preferred Reporting Items for Systematic Reviews and Meta‐Analysis guidelines, and risk of bias was assessed using the Methodological Index for Non‐Randomized Studies.

**Results:**

Sixty‐five studies comprising 2194 patients were analyzed—29 from APAC (803 patients) and 36 from NA (1391 patients). Patients’ age (APAC: 9‐18 years; NA: 6‐19 years) and follow‐up duration (APAC: 8‐184 months; NA: 2‐216 months) were similar. Significant regional differences were observed across multiple domains. Sex distribution was markedly skewed in APAC, where most studies included predominantly male participants (91.9%) without intentional female disaggregation, compared with a more balanced cohort in NA (55.3%). Although the elbow was the most common OCD site overall, knee involvement was significantly higher in NA (36.1%) than APAC (20.7%). Imaging modalities differed—radiographs were the most common imaging tool in both regions, but magnetic resonance imaging was more frequently used in NA (48.9%) compared with APAC (35.8%). Surgical approaches also varied significantly: osteochondral grafting was more common in APAC (41.7%), whereas arthroscopic techniques were favored in NA (73.5%). In situ fixation rates were similar between regions. Standardized reporting and prospective, multicenter registries are essential for enabling patient‐level analysis and guiding region‐specific, evidence‐based care for pediatric OCD.

**Conclusions:**

There are significant regional variations in epidemiology, imaging modalities, and surgical management for pediatric OCD. The predominantly male bias in the APAC region, greater use of magnetic resonance imaging, and arthroscopic techniques in the NA region reflect underlying biological, cultural, and health care‐related differences.

**Level of Evidence:**

Level IV, systematic review of Level III and IV studies.

## Graphical abstract

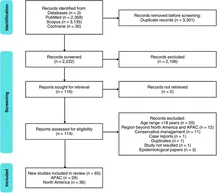

Osteochondritis dissecans (OCD) is defined as a focal idiopathic lesion of subchondral bone which is potentially reversible but at risk of chondral instability and disruption of articular cartilage, potentially resulting in premature osteoarthritis.^1^ OCD can affect both the juvenile and adult population; however, juvenile OCD is associated with a better prognosis and is more common with an incidence of 11.2 per 100,000.^2^ OCD typically affects the knee, elbow, and ankle joints, with the knee being the most commonly involved site. Generally, men have a higher risk of OCD in the knee and elbow joints (4 and 7 times, respectively), whereas women have a 1.5 times higher risk of developing ankle OCD specifically in the talus.^2^, ^3^, ^4^ OCD initially presents with joint pain that worsens with physical activity. As the condition progresses, mechanical symptoms such as catching, locking, or restricted range of motion may develop. The initial evaluation typically includes radiographs; however, magnetic resonance imaging (MRI) remains the gold standard for diagnosis.

Management strategies for OCD can be conservative or surgical, depending on skeletal maturity, and instability of the lesion is characterized by the presence of an articular cartilage fracture and subchondral bone separation. The primary goals of treatment are twofold: a pain‐free, mobile joint and preservation of the articular surface.^5^ Common interventions include platelet‐rich plasma injections, arthroscopic drilling (including microfracturing), osteochondral graft transplants, bioabsorbable fixation, or a combination.^6^, ^7^, ^8^, ^9^ However, the 2010 clinical practice guidelines from the American Academy of Orthopaedic Surgeons highlighted a lack of high‐level evidence for guiding OCD management.^10^

There is a notable gap in the existing literature regarding regional variations in the epidemiology and management of pediatric OCD. Although individual studies have evaluated diagnostic and surgical techniques and outcomes, there is limited data comparing the epidemiology, diagnostic approaches, and surgical interventions for pediatric OCD between Asia‐Pacific (APAC) and North America (NA). Identifying variations in sex distribution, age groups affected, and site of OCD lesions allows for the development of targeted public health strategies and early detection initiatives tailored to each region. Furthermore, comparing diagnostic and treatment strategies can highlight the most effective and practical options suited to each region, informing region‐specific guidelines and improving clinical outcomes for pediatric patients with OCD. Such comparisons may also guide future research efforts by highlighting existing knowledge gaps and supporting the development of regionally appropriate interventions of pediatric OCD.

The purpose of this systematic review was to evaluate regional variations in epidemiology, diagnostic approaches, and surgical interventions for pediatric OCD between APAC and NA. The hypothesis was that there are significant regional differences in these aspects between APAC and NA.

## METHODS

A comprehensive literature search using the PubMed, Scopus, and Cochrane Library databases was undertaken in August 2025 with the following search string: (“pediatric” OR “paediatric” OR “child” OR “youth” OR “juvenile”) AND (“osteochondritis” OR “dissecans” OR “cartilage injury”). Each keyword is connected via a Boolean operator “AND” or “OR.”

All abstracts were screened and the relevant manuscripts reviewed. Bibliographies of relevant published papers were also reviewed to identify any other relevant studies not captured by the primary search. Articles were screened for the following criteria: original research, pediatric population 18 years old or younger, studies reporting on surgical interventions, and OCD of any joint (knee/elbow/ankle) were included. Articles not written in English, case reports, review papers with no original data, incomplete data, or trials still in progress were excluded. No time limit was given for the publication date.

This research was conducted in accordance with the Preferred Reporting Items for Systematic Reviews and Meta‐Analysis guidelines.^11^ The study selection process involved a 2‐staged process as shown in the Preferred Reporting Items for Systematic Reviews and Meta‐Analysis flow diagram in Figure 1. In the first instance, the title and abstracts of each study identified by the search terms were screened for eligibility using the abovementioned inclusion and exclusion criteria independently by 2 authors (J.R.Y.W., Y.X.L.). Second, the full texts of the potentially eligible studies including the reference list of publications were screened for additional studies that met the inclusion criteria.

**FIGURE 1:**
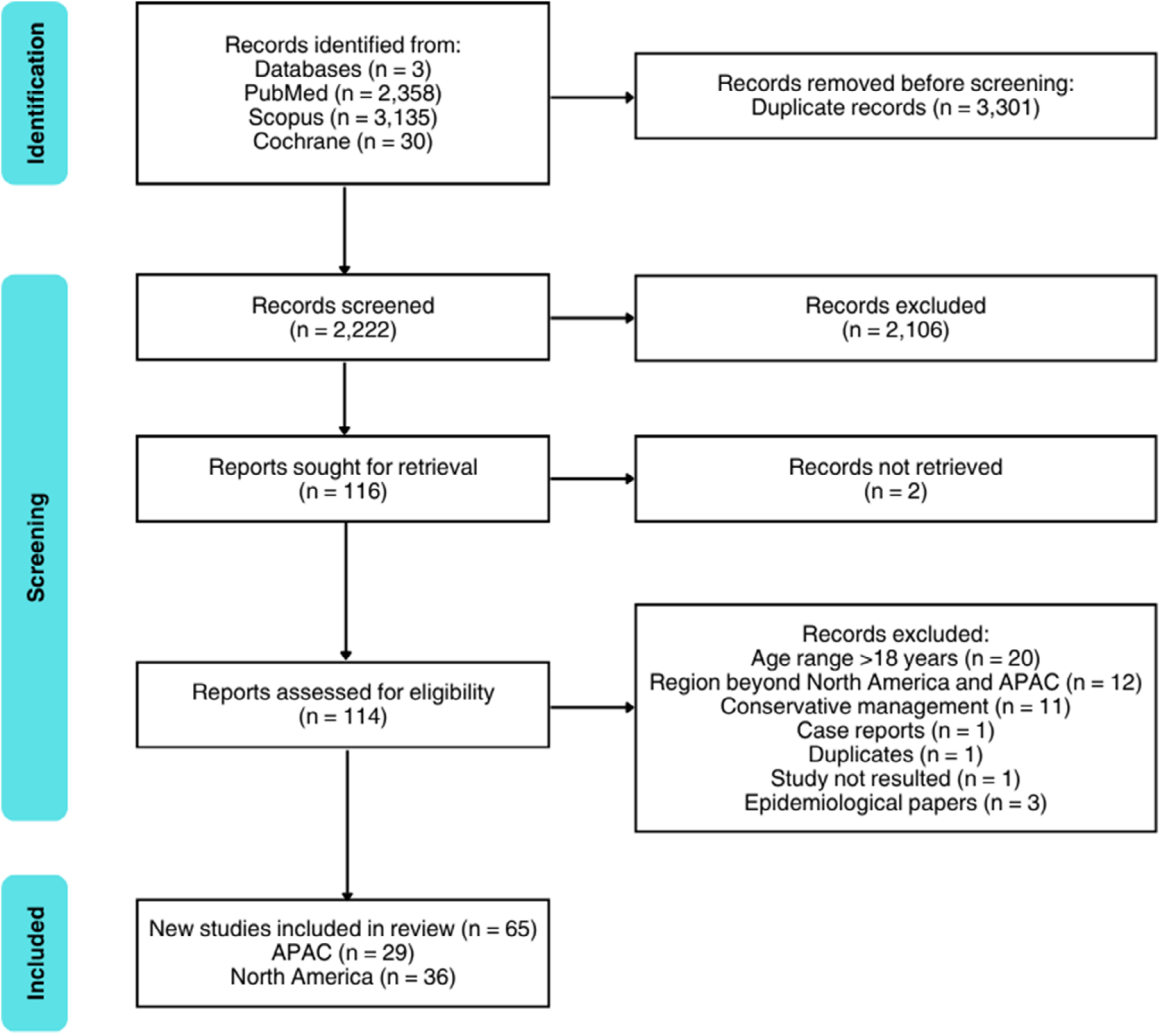
PRISMA flow diagram. (APAC, Asia‐Pacific; PRISMA, Preferred Reporting Items for Systematic Reviews and Meta‐Analysis.)

Details of each study including the state or country of origin, sample size, mean patient age with standard deviation (SD) and range, sex distribution, anatomical site of OCD, preoperative imaging modalities, surgical interventions, follow‐up duration, and level of evidence were retrieved by 2 authors (J.R.Y.W., Y.X.L.) and documented in Table 1. For studies that failed to report SD values for continuous data such as patient age and follow‐up duration, the SD was approximated from the provided ranges using the method described by Hozo et al.^12^

**TABLE 1:** Summary of All Included APAC and NA Studies

** Author**	** Year Published**	** Study Design**	** Level of Evidence**	** Region**	** Country**	** No. of Lesions**	** Age Mean, yr**	** Age SD, yr**	** Age Range, yr**	** Sex (F)**	** Sex (M)**	** OCD Site**	** MRI**	** CT**	** Radiograph**	** Arthroscopic Techniques**	** Osteochondral Graft**	** Internal Fixation**	** Follow‐Up Mean, mo**	** Follow‐Up SD, mo**	** Follow‐Up Range, mo**
Adachi^13^	2009	Retrospective case series	IV	APAC	Japan	20	12	3	9‐15	2	10	Knee	20	0	20	20	0	0	32.4	NR	NR
Adachi^14^	2015	Retrospective case series	IV	APAC	Japan	33	14.4	1.5	11‐17	7	23	Knee	33	0	33	15	0	18	39.6	12.6	25.2‐75.6
Arai^15^	2019	Retrospective case series	IV	APAC	Japan	7	13.3	1	12‐14	0	7	Elbow	0	0	7	7	0	0	36.1	NR	24‐68
Chen^16^	2015	Retrospective case series	IV	APAC	China	63	13.7	1.25	11‐16	34	29	Knee	63	0	63	63	0	0	56.4	15.9	24‐87.6
Funakoshi^17^	2018	Prospective case series	IV	APAC	Japan	22	13.5	1.2	NR	0	22	Elbow	22	0	22	0	22	0	27.5	6	24‐48
Imada^18^	2021	Prospective case series	IV	APAC	Japan	10	13.5	2	12‐16	0	10	Elbow	10	0	10	0	0	10	26.7	NR	NR
Iwasaki^19^	2006	Prospective case series	IV	APAC	Japan	8	14	2.5	11‐16	0	8	Elbow	8	0	8	0	8	0	24	NR	14‐42
Iwasaki^20^	2009	Prospective case series	IV	APAC	Japan	10	13.7	2.5	12‐17	0	10	Elbow	10	0	0	0	10	0	12	NR	NR
Kawasaki^21^	2003	Retrospective case series	IV	APAC	Japan	16	12.5	4.5	9‐18	3	9	Knee	16	0	16	16	0	0	16	3	12‐24
Kiyomatsu^22^	2021	Retrospective cohort study	III	APAC	Japan	34	13.9	1	12‐16	NR	NR	Elbow	34	0	34	0	0	34	28.7	6.5	24‐50
Kosaka^23^	2013	Retrospective case series	IV	APAC	Japan	32	14.4	2	10‐18	0	32	Elbow	32	0	32	0	19	13	58.6	30.5	24‐146
Maruyama^24^	2016	Retrospective case series	IV	APAC	Japan	10	12.3	2.5	10‐15	0	10	Elbow	10	0	10	0	10	0	25	NR	10‐52
Maruyama^25^	2014	Retrospective case series	IV	APAC	Japan	33	13.6	1.5	11‐17	0	33	Elbow	33	0	33	0	33	0	28.4	16	12‐76
Matsuura^26^	2017	Retrospective cohort study	III	APAC	Japan	87	13.2	0.83	11‐16	1	86	Elbow	87	0	87	0	87	0	43	12.67	24‐100
Matsuura^27^	2020	Retrospective case series	IV	APAC	Japan	23	14.7	1	13‐17	0	23	Elbow	0	0	23	23	0	0	138	9	120‐156
Mihara^28^	2010	Retrospective case series	IV	APAC	Japan	27	13.3	1.5	10‐16	0	27	Elbow	17	0	27	7	7	13	37.4	NR	NR
Miyake^29^	2011	Retrospective case series	IV	APAC	Japan	106	15	1	12‐18	1	105	Elbow	0	0	106	106	0	0	13	6.33	8‐46
Nishimura^30^	2011	Prospective case series	IV	APAC	Japan	12	14.4	2.5	12‐17	0	12	Knee	0	0	12	0	12	0	34.1	NR	NR
Nishinaka^31^	2014	Retrospective case series	IV	APAC	Japan	22	13.9	1.25	11‐16	NR	NR	Elbow	22	0	22	0	22	0	27	16.25	12‐77
Oshiba^32^	2016	Prospective case series	IV	APAC	Japan	11	14	1.5	13‐16	0	11	Elbow	0	0	11	0	11	0	33.9	NR	24‐59
Shimada^33^	2017	Prospective case series	IV	APAC	Japan	16	14.4	1.25	12‐17	1	15	Elbow	16	0	16	0	16	0	31	10.5	24‐66
Takeba^34^	2015	Retrospective case series	IV	APAC	Japan	13	14	2	12‐16	NR	NR	Elbow	13	13	13	5	0	13	24	NR	12‐50
Uchida^35^	2015	Prospective case series	IV	APAC	Japan	18	14.2	1	12‐16	0	18	Elbow	18	18	18	18	0	18	39	3.5	36‐50
Ueda^36^	2017	Prospective case series	IV	APAC	Japan	38	14	0.5	13‐15	5	33	Elbow	0	14	14	14	0	0	96	21	60‐144
Ueda^37^	2021	Retrospective cohort study	III	APAC	Japan	48	14	0.5	13‐15	2	46	Elbow	0	48	48	19	29	0	88.8	24	60‐156
Ueki^38^	2019	Prospective case series	IV	APAC	Japan	17	11.8	0.25	11‐12	0	17	Elbow	0	0	17	0	0	16	70.1	39.75	25‐184
Yamagami^39^	2018	Retrospective cohort study	III	APAC	Japan	30	14	1.25	12‐17	0	30	Elbow	0	0	30	0	30	0	37	21.25	14‐99
Yamamoto^40^	2006	Retrospective case series	IV	APAC	Japan	18	13.6	1.5	10‐16	0	18	Elbow	18	0	18	0	18	0	42	9.75	24‐63
Yonetani^41^	2012	Retrospective case series	IV	APAC	Japan	19	12	0.75	11‐14	2	16	Knee	0	19	0	19	0	0	30	6	24‐48
Backes^42^	2014	Retrospective case series	IV	NA	Ohio	59	11.8	2.75	6‐17	6	22	Knee	59	0	59	35	0	9	NR	NR	NR
Bae^43^	2020	Retrospective case series	IV	NA	Massachusetts	28	14.2	3.15	NR	14	14	Elbow	28	0	28	14	8	0	6.3	5.5	5‐27
Baghdadi^7^	2022	Retrospective case series	IV	NA	Pennsylvania	139	12.7	1.5	9‐18	37	102	Knee	139	0	0	139	0	0	17.8	13.2	NR
Boughanem^44^	2011	Retrospective case series	IV	NA	Illinois	34	12.6	2	8‐16	10	21	Knee	34	0	34	34	0	0	48	16.5	18‐84
Bram^45^	2025	Retrospective case series	IV	NA	New York	20	12.8	1.4	NR	15	5	Elbow	NR	NR	NR	20	20	0	22.2	13	12.7‐65.1
Broughton^46^	2021	Retrospective case series	IV	NA	Washington	59	14	NR	10‐18	21	38	Elbow	NR	NR	NR	50	9	0	72	60	12‐216
Byrd^47^	2002	Retrospective cohort study	III	NA	Tennessee	10	13.8	1.25	11‐16	0	10	Elbow	0	0	10	10	0	0	46.8	NR	NR
Donaldson^48^	2008	Retrospective case series	IV	NA	Michigan	16	12.3	1.5	9‐15	7	8	Knee	16	0	0	16	0	0	21	7.5	8‐38
Edmonds^49^	2010	Retrospective case series	IV	NA	Florida	59	13.4	2.65	8‐18.6	15	36	Knee	59	0	59	59	0	0	36.3	17.68	1.3‐72
Fabricant^50^	2018	Retrospective case series	IV	NA	Massachusetts	15	12.7	0.625	11.7‐14.2	4	11	Knee	15	0	15	15	0	15	12	NR	6.0‐19.2
Hennrikus^51^	2014	Retrospective case series	IV	NA	Massachusetts	26	14.1	1.7	NR	13	13	Elbow	22	0	26	26	0	26	39	21	12‐96
Heyworth^52^	2023	Randomized controlled trial	I	NA	Massachusetts	91	12.3	1.34	9‐15	32	59	Elbow	91	0	91	91	0	0	NR	NR	NR
Husen^53^	2022	Retrospective case series	IV	NA	Minnesota	16	14.9	0.6	13.8‐16.2	4	12	Knee	0	0	16	16	0	16	42.3	12.7	1.7‐52.5
Jackson^54^	1989	Retrospective case series	IV	NA	Ohio	10	13.3	1.75	10‐17	7	0	Elbow	0	0	10	9	0	0	34.8	NR	7‐86
Johnson^55^	2021	Retrospective case control	III	NA	Pennsylvania	74	13.1	2.7	7.1‐18	45	29	Ankle	0	0	74	58	3	5	NR	NR	NR
Jones^56^	2010	Retrospective case series	IV	NA	Washington	22	13.1	1.75	10‐17	7	14	Elbow	22	0	0	10	12	0	48	15.5	21‐83
Kocher^57^	2007	Retrospective case series	IV	NA	Massachusetts	26	14.7	1.25	11‐16	11	13	Knee	0	0	26	0	0	26	51	38.25	24‐177
Kocher^58^	2001	Retrospective case series	IV	NA	Massachusetts	30	12.3	1.9	8.5‐16.1	8	15	Knee	0	0	30	30	0	0	46.8	16.1	24‐86.4
Kramer^59^	2015	Retrospective case series	IV	NA	Massachusetts	109	14.3	2.3	7‐18	75	25	Ankle	109	0	109	86	0	22	39.6	19.6	12‐129.6
Kramer^60^	2015	Retrospective case series	IV	NA	Massachusetts	29	14.7	2.1	9‐18	9	17	Knee	29	0	29	21	0	8	45.6	24.75	9‐108
Lewine^61^	2016	Retrospective case series	IV	NA	Massachusetts	21	13.4	1.87	NR	8	13	Elbow	18	0	21	21	0	0	26.4	14.28	NR
Obey^62^	2021	Retrospective case series	IV	NA	Missouri	31	12.9	1.75	9‐16	12	19	Elbow	NR	NR	NR	25	6	0	6.1	2.9	NR
Pan^63^	2025	Retrospective case series	IV	NA	Pennsylvania	12	14.2	2.1	NR	9	3	Ankle	5	2	12	12	0	4	23.1	NR	3‐58
Ruch^64^	1998	Retrospective case series	IV	NA	North Carolina	12	14.5	1.5	11‐17	2	10	Elbow	0	0	12	12	0	0	38.4	NR	2.2‐5.9
Shi^65^	2012	Retrospective case series	IV	NA	Massachusetts	42	14.2	1.75	11.2‐18.2	16	26	Elbow	42	0	0	42	0	6	19.5	15.03	3.5‐63.6
Tabaddor^66^	2010	Retrospective case series	IV	NA	Massachusetts	24	14.4	2	10‐18	10	14	Knee	24	0	24	19	0	24	22.4	NR	NR
Tan^67^	2022	Retrospective case series	IV	NA	Canada	19	14.6	2.1	11‐18	18	1	Ankle	3	5	11	19	0	0	14.8	11.7	2.4‐47.6
Tis^68^	2012	Retrospective case series	IV	NA	California	13	13.1	1.07	10.8‐14.6	5	7	Elbow	13	0	13	13	0	0	23.4	16.7	2‐60
Tisano^69^	2022	Prospective cohort study	III	NA	Texas	37	15.4	2	9.6‐17.6	15	22	Knee	37	0	37	0	37	0	25.2	12.9	12‐63.6
Tivnon^70^	1976	Retrospective case series	IV	NA	California	13	14	1.25	12‐17	0	12	Elbow	0	0	13	13	0	0	45	NR	12‐96
Wall^71^	2014	Retrospective case series	IV	NA	Massachusetts	24	14	2	10‐18	4	17	Elbow	24	24	24	12	0	6	36	44.8	12‐144
Wang^72^	2019	Retrospective case series	IV	NA	Massachusetts	28	13.4	1.6	NR	15	13	Elbow	28	0	28	4	0	1	13	8	NR
Webb^73^	2013	Retrospective case series	IV	NA	Minnesota	20	14.5	1.25	12‐17	6	14	Knee	0	0	20	5	6	18	84	33	24‐156
Wulf^74^	2012	Retrospective case series	IV	NA	Minnesota	10	13.9	1.925	10.8‐18.5	4	6	Elbow	10	0	10	10	0	0	42	NR	27‐54
Yehyawi^75^	2024	Retrospective case series	IV	NA	Indiana	40	12.1	NR	9‐14	30	1	Elbow	NR	NR	NR	40	0	0	62.4	22.5	24‐114
Zheng^76^	2024	Prospective cohort study	III	NA	Massachusetts	173	13.7	2	NR	83	71	Elbow	173	0	173	43	63	21	15.6	12	NR

APAC, Asia‐Pacific; CT, computed tomography; F, female; M, male; mo, months; MRI, magnetic resonance imaging; NA, North America; NR, not recorded; OCD, osteochondritis dissecans; SD, standard deviation; yr, years.

A summary and comparison of the APAC and NA studies of the above parameters are shown in Table 2. For the avoidance of doubt, the APAC region encompassed countries in East Asia including China, Japan, and the Republic of Korea; Southeast Asia; South Asia; and Oceania and Australia. The NA region was defined as including the United States, Canada, and Mexico. For papers in which the location of data collection was unclear, the region was classified based on the institutional affiliation of the first author. Attempts were made to contact corresponding authors of the studies where clarification of details was necessary.

**TABLE 2:** Comparison of APAC and NA Studies on the Region, Level of Evidence, Sample Size, Patient Demographics (Sex, Age), Anatomical Site of OCD, Preoperative Imaging Modalities, Surgical Interventions, and Follow‐Up Duration

	**APAC (n = 29)**	**NA (n = 36)**
Region (%)	Japan: 28 (96.6%)	United States: 35 (97.2%)
	China: 1 (3.4%)	Canada: 1 (2.8%)
Level of evidence (%)	Level I: 0 (0%)	Level I: 1 (2.8%)
	Level II: 0 (0%)	Level II: 0 (0%)
	Level III: 4 (13.8%)	Level III: 4 (11.1%)
	Level IV: 25 (86.2%)	Level IV: 31 (86.1%)
Total sample size (no. of OCD lesions)	803	1391
Sex (%)	Female: 58 (8.1%)	Female: 577 (44.7%)
	Male: 680 (91.9%)	Male: 713 (55.3%)
Age range, yr	9‐18	6‐18.5
Site of OCD	Knee: 6 (20.7%)	Knee: 13 (36.1%)
	Elbow: 23 (79.3%)	Elbow: 19 (52.8%)
	Ankle: 0 (0%)	Ankle: 4 (11.1%)
Preoperative imaging		
(1) MRI	482 (35.8%)	1000 (48.9%)
(2) CT	112 (8.3%)	31 (1.5%)
(3) Radiographs	750 (55.8%)	1014 (49.6%)
Surgical interventions		
(1) Arthroscopic techniques	332 (41.4%)	1029 (73.5%)
(2) Osteochondral graft transplant	334 (41.7%)	164 (11.7%)
(3) In situ fixation	135 (16.9%)	207 (14.8%)
Follow‐up duration range, mo	8‐184	1.3‐216

*Note*: n, sample size.

APAC, Asia‐Pacific; CT, computed tomography; mo, months; MRI, magnetic resonance imaging; NA, North America; OCD, osteochondritis dissecans; yr, years.

The studies included were examined for study design. The revised Methodological Index for Non‐Randomized Studies (MINORS) tool is used to determine the methodological quality of the studies.^77^ The items are scored 0 if not reported, 1 if reported but inadequate, and 2 if reported and adequate. The global ideal score is 24 for case‐control studies and 16 for case series.^77^ The quality of study assessment was performed independently by 2 authors (J.R.Y.W., Y.X.L.). Any disagreements were resolved through a consensus discussion between the rest of the senior authors (S.H.S.T., J.H.P.H., M.W.).

Categorical outcomes such as the origin of the study, sex, site of OCD, preoperative imaging, and surgical interventions were primarily evaluated qualitatively because of the lack of variance data and substantial heterogeneity among the included studies. Furthermore, the potential risk of bias precluded appropriate quantitative data pooling for meta‐analysis.

As for continuous outcomes such as age and follow‐up duration, individual study results are graphically represented in a forest plot without the summary estimate. To account for the variability in sample sizes across studies, 95% confidence intervals were calculated using the Student's t‐distribution. Data analysis was performed using R statistical software (version 4.5.1, R Foundation for Statistical Computing, Vienna, Austria).

## RESULTS

A total of 5523 manuscripts were identified through searching the 3 databases. Of these, 65 studies met the inclusion criteria for this systematic review, comprising 29 studies from the APAC region^13^, ^14^, ^15^, ^16^, ^17^, ^18^, ^19^, ^20^, ^21^, ^22^, ^23^, ^24^, ^25^, ^26^, ^27^, ^28^, ^29^, ^30^, ^31^, ^32^, ^33^, ^34^, ^35^, ^36^, ^37^, ^38^, ^39^, ^40^, ^41^ and 36 studies from NA.^7^, ^42^, ^43^, ^44^, ^45^, ^46^, ^47^, ^48^, ^49^, ^50^, ^51^, ^52^, ^53^, ^54^, ^55^, ^56^, ^57^, ^58^, ^59^, ^60^, ^61^, ^62^, ^63^, ^64^, ^65^, ^66^, ^67^, ^68^, ^69^, ^70^, ^71^, ^72^, ^73^, ^74^, ^75^, ^76^ The details of each study are summarized in Table 1.

Of the 29 APAC studies, 28 (96.6%) originated from Japan, with only 1 study (3.4%) from China. Among the 36 NA studies, a single study (2.8%) originated from Canada, whereas the remaining 35 studies (97.2%) originated across 16 US states, with the largest proportion (14 out of 35 studies, 40%) originating from Massachusetts. The geographical representation of the included studies is shown in Figure 2.

**FIGURE 2:**
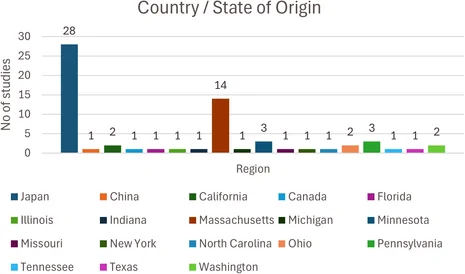
Diagram showing the geographical distribution of the included studies.

The total sample size comprised 2194 patients, of which 803 and 1391 were from the APAC and NA region, respectively. As summarized in Table 2, the ages of the patients from the 2 regions were similar, ranging from 9 to 18 years old in APAC and 6 to 18.5 years old in NA. However, multiple studies did not document the age range of the participants included in their studies.^17^, ^43^, ^45^, ^51^, ^61^, ^63^, ^72^, ^76^ Ninety‐five percent confidence intervals were calculated in studies that reported the mean age and SD of their patients. These individual study results are graphically presented in a forest plot to allow for a visual comparison of results without pooling of data, as shown in Figure 3. It should be noted that only studies with calculated 95% confidence intervals were plotted with points and error bars. Studies with missing data were represented in the forest plot but without error bars.

**FIGURE 3:**
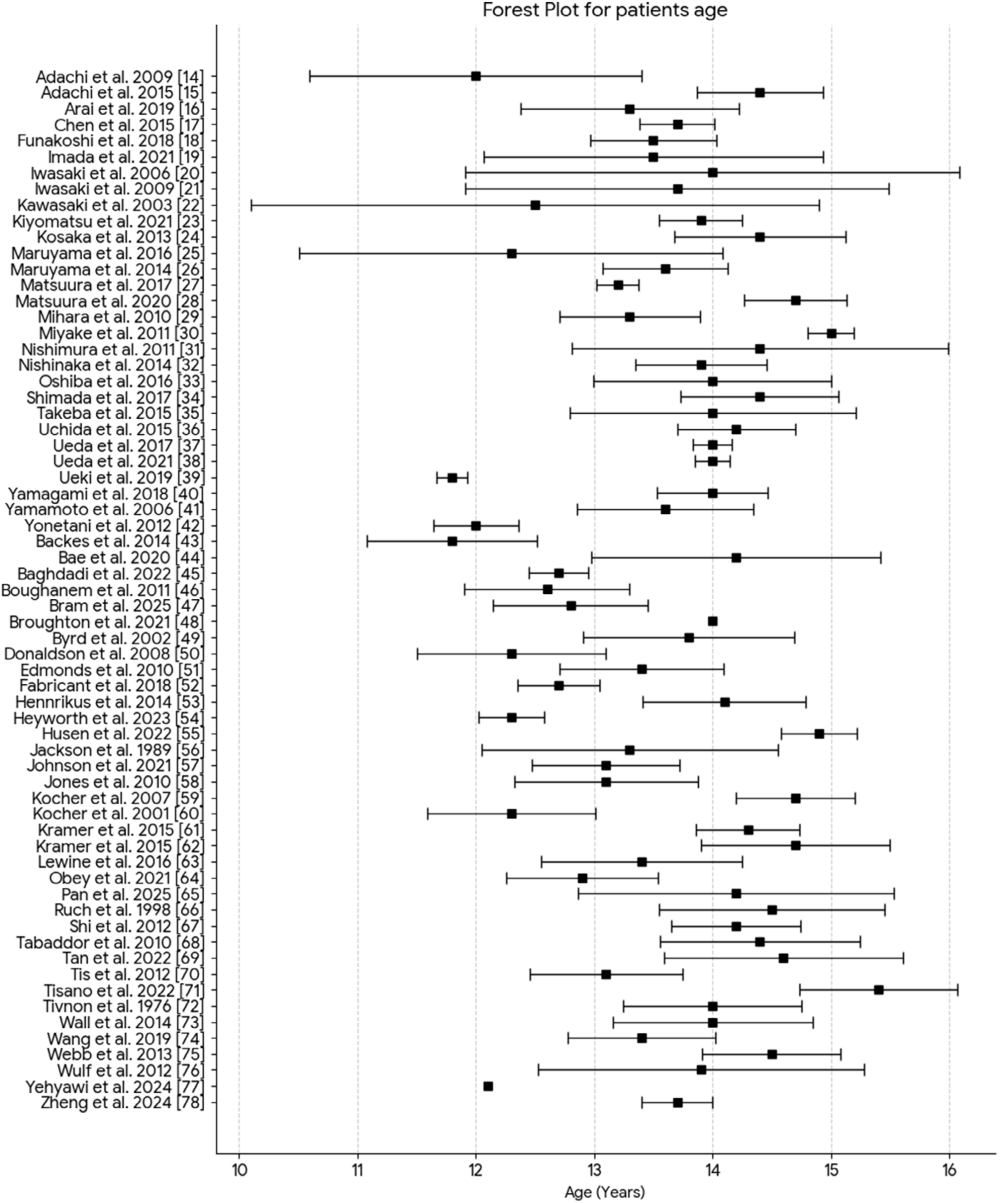
Forest plot of the patients’ age in the included studies.

Comparative analysis of the APAC and NA studies revealed significant regional variation across several domains. Sex distribution differed with males predominating in the APAC cohort (680 out of 738, 91.9%), whereas NA studies showed a more balanced distribution with 55.3% (713 out of 1290) males, as illustrated in Figure 4. Notably, none of the 65 studies intentionally disaggregated data by sex. However, the gender of patients was not reported in 3 studies by Kiyomatsu et al.,^22^ Nishinaka et al.,^31^ and Takeba et al.^34^ and hence were excluded in calculations of sex distribution.

**FIGURE 4:**
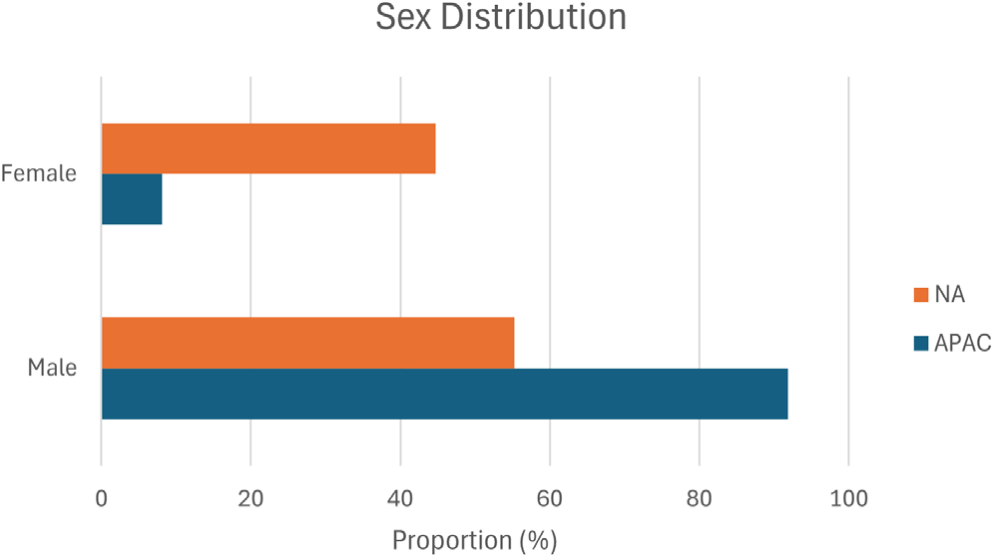
Diagram showing the sex distribution of patients in the included studies. (APAC, Asia‐Pacific; NA, North America.)

The most common anatomical site of OCD was found in the elbow, followed by the knee and least commonly in the ankle in both regions. However, the data showed regional variation, with knee lesions occurring nearly twice as frequently in NA (13 out of 36, 36.1%) compared with APAC (6 out of 29, 20.7%), as shown in Figure 5.

**FIGURE 5:**
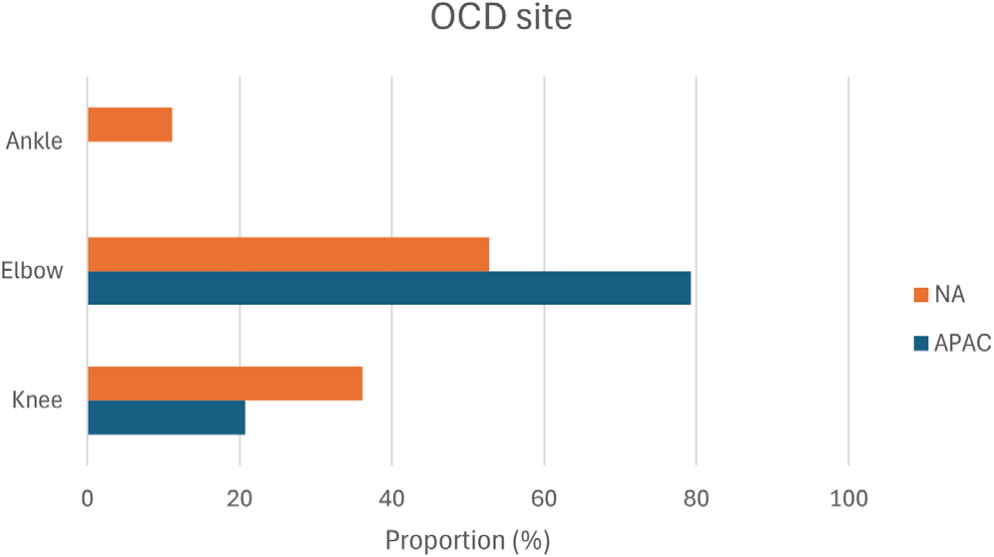
Bar plot diagram showing the distribution of OCD sites in the included studies. (APAC, Asia‐Pacific; NA, North America; OCD, osteochondritis dissecans.)

Preoperative imaging modalities also varied significantly in both regions. The most common imaging tool in the APAC studies was radiographs where 750 out of 1344 (55.8%) of studies reported its use, whereas in NA, radiographs were used in 1014 out of 2045 (49.6%) of studies, followed closely by MRI in 48.9% (1000 out of 2045). The use of computed tomography was more frequently reported in APAC 8.3% (112 out of 1344) compared with 1.5% (31 out of 1344) of NA studies, as shown in Figure 6.

**FIGURE 6:**
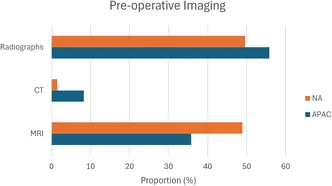
Diagram showing the distribution of the different imaging modalities used in the included studies. (APAC, Asia‐Pacific; CT, computed tomography; MRI, magnetic resonance imaging; NA, North America.)

There were distinct regional differences when it came to the type of surgical interventions. Three hundred and thirty‐four of 801 APAC studies favored osteochondral grafts (41.7%) compared with only 11.7% (164 out of 1400) in the NA region. However, there was a greater trend toward arthroscopic techniques including debridement, microfracturing, or drilling in 1029 of the 1400 (73.5%) NA studies compared with just 332 out of 801 (41.4%) APAC studies. In situ fixation using various types of metallic screws or bioabsorbable implants were similarly frequently reported across the 2 regions with 135 out of 801 (16.9%) APAC studies and 207 out of the 1400 (14.8%) NA studies. All the above findings are summarized in Figure 7.

**FIGURE 7:**
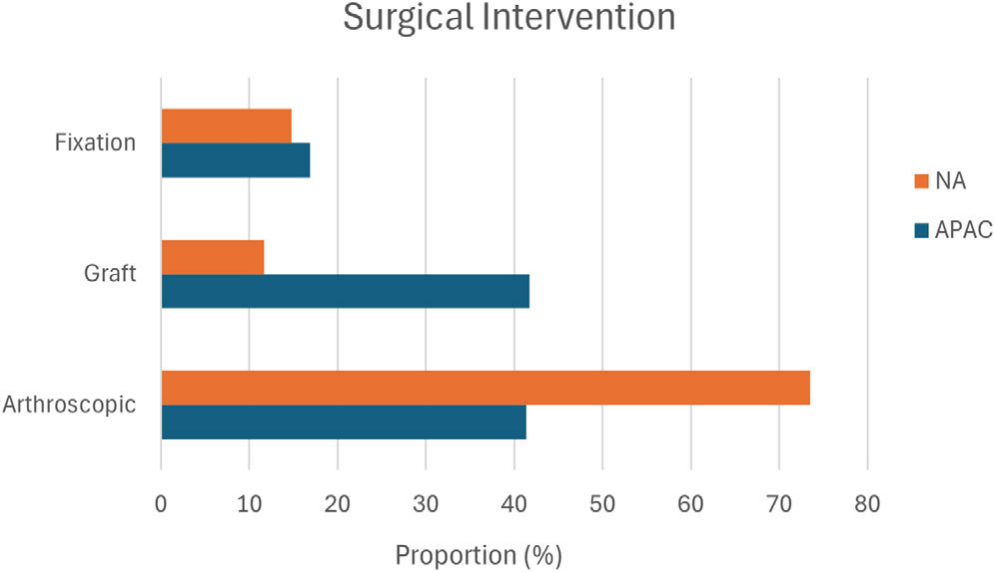
Diagram showing the distribution of surgical interventions used in the included studies. (APAC, Asia‐Pacific; NA, North America.)

Both NA and APAC regions had similar follow‐up duration. The range of follow‐up duration varied greatly intraregionally with studies following up patients for a minimum of 8 months to a maximum of 184 months in APAC and a minimum of 1.3 months to a maximum of 18 years in NA. However, it should be noted that multiple studies from both regions failed to report the range in follow‐up duration.^7^, ^13^, ^18^, ^20^, ^28^, ^30^, ^42^, ^47^, ^52^, ^55^, ^61^, ^62^, ^66^, ^72^, ^76^ Nevertheless, the individual study results are graphically presented in a forest plot to allow for a visual comparison of results without pooling of data, as shown in Figure 8.

**FIGURE 8:**
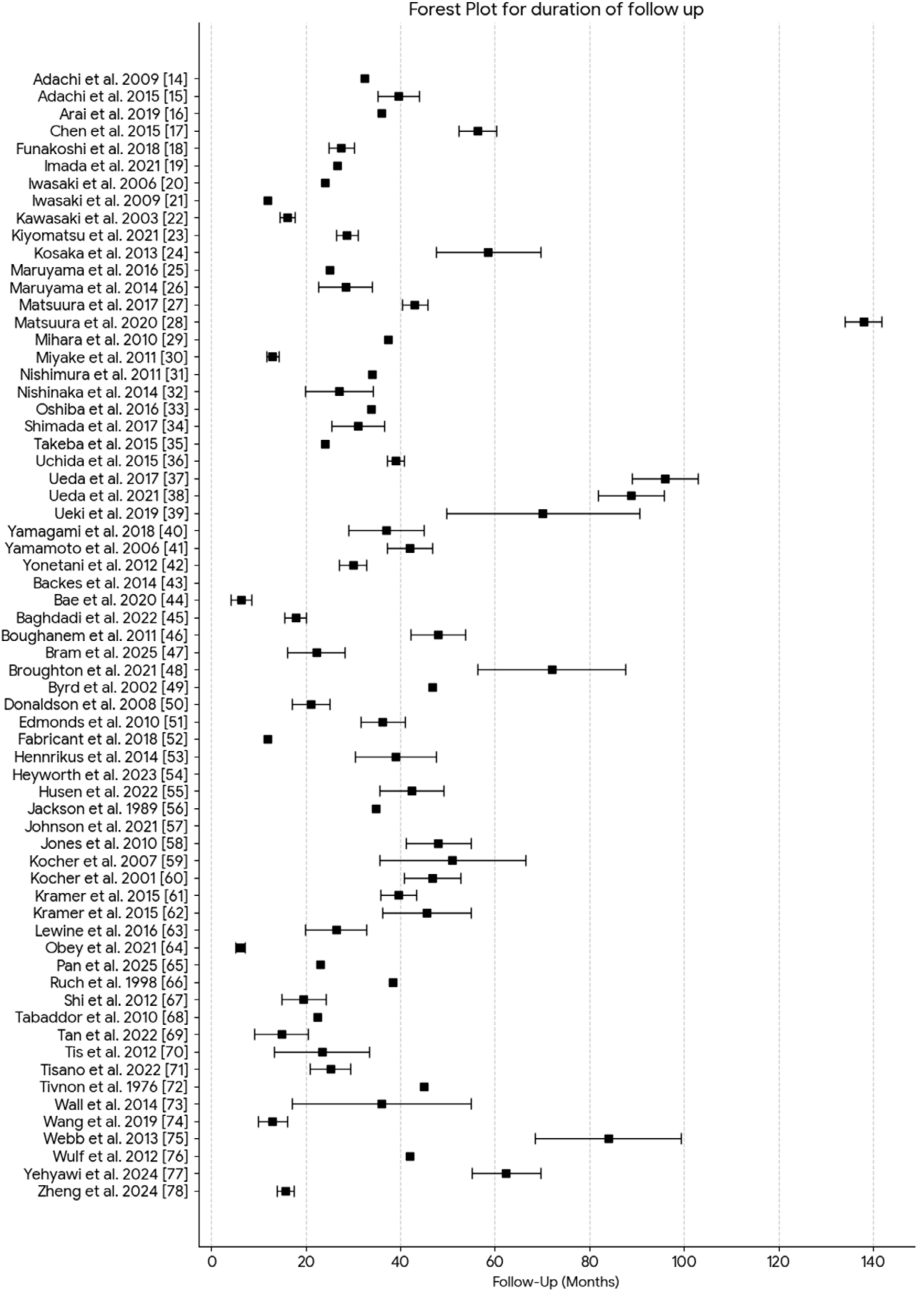
Forest plot of the follow‐up duration in the included studies.

The studies included from both APAC and NA regions had comparable levels of evidence. Four of the 29 (13.8%) APAC studies were Level III evidence, whereas the rest of the 25 studies (86.2%) were Level IV evidence. Similarly, 4 of the 36 (11.1%) NA studies were Level III evidence, and 31 of the 36 (86.1%) were Level IV evidence. These are summarized in Table 2.

The quality of the studies included in this systematic review was evaluated using the revised MINORS tool^12^ and was found to be comparable across regions. The average MINORS score for comparative studies was 16/24 in the APAC region and 16.6/24 in NA. For noncomparative studies, the average MINORS score was 10.3/16 in APAC and 10.4/16 in NA. An assessment of the quality of all included studies is presented in Tables 3 and 4.

**TABLE 3:** Summary of MINORS Score for APAC Studies

** Study**		** For All Nonrandomized Studies (Level III‐IV Evidence)**								** Additional Items for Comparative Studies Only**				
** Author**	** Year Published**	** Clearly Stated Aim**	** Consecutive Patient Inclusion**	** Prospective Data Collection**	** Endpoints Appropriate to Aim**	** Unbiased Assessment of Endpoint**	** Follow‐Up Period Appropriate**	** Loss to Follow‐Up <5%**	** Prospective Calculation of the Study Size**	** Adequate Control Group**	** Contemporary Groups**	** Baseline Equivalence of Groups**	** Adequate Statistical Analysis**	** Total**
Adachi^13^	2009	2	2	0	2	0	2	2	0	NA	NA	NA	NA	10/16
Adachi^14^	2015	2	2	0	1	0	2	2	0	NA	NA	NA	NA	8/16
Arai^15^	2019	2	2	0	2	0	2	2	0	NA	NA	NA	NA	10/16
Chen^16^	2015	2	2	0	2	0	2	2	0	NA	NA	NA	NA	11/16
Funakoshi^17^	2018	2	2	2	2	0	2	2	0	NA	NA	NA	NA	14/16
Imada^18^	2021	2	2	2	2	0	2	2	0	NA	NA	NA	NA	12/16
Iwasaki^19^	2006	2	2	2	2	0	2	2	0	NA	NA	NA	NA	12/16
Iwasaki^20^	2009	2	2	2	2	2	2	2	0	NA	NA	NA	NA	14/16
Kawasaki^21^	2003	0	1	0	2	0	2	2	0	NA	NA	NA	NA	7/16
Kiyomatsu^22^	2021	1	2	0	2	0	2	2	0	NA	NA	NA	NA	10/16
Kosaka^23^	2013	2	2	0	2	0	2	0	0	NA	NA	NA	NA	8/16
Maruyama^24^	2016	2	2	0	2	0	2	2	0	NA	NA	NA	NA	10/16
Maruyama^25^	2014	2	2	0	2	0	2	2	0	NA	NA	NA	NA	10/16
Matsuura^26^	2017	2	2	0	2	0	2	2	0	NA	NA	NA	NA	10/16
Matsuura^27^	2020	2	2	0	2	2	2	2	0	NA	NA	NA	NA	12/16
Mihara^28^	2010	2	2	0	2	0	2	2	0	NA	NA	NA	NA	10/16
Miyake^29^	2011	2	2	0	2	0	2	2	0	NA	NA	NA	NA	10/16
Nishimura^30^	2011	2	2	2	2	0	1	2	0	NA	NA	NA	NA	12/16
Nishinaka^31^	2014	2	2	0	2	0	2	2	0	NA	NA	NA	NA	10/16
Oshiba^32^	2016	2	2	0	2	0	2	2	0	NA	NA	NA	NA	10/16
Shimada^33^	2017	2	2	0	2	0	2	2	0	NA	NA	NA	NA	10/16
Takeba^34^	2015	2	2	0	2	0	1	2	0	NA	NA	NA	NA	10/16
Uchida^35^	2015	2	2	2	2	0	2	2	0	NA	NA	NA	NA	12/16
Ueda^36^	2017	2	2	0	2	0	2	1	0	NA	NA	NA	NA	9/16
Ueda^37^	2021	2	2	0	2	0	2	2	0	NA	NA	NA	NA	10/16
Ueki^38^	2019	2	2	0	2	0	2	1	0	NA	NA	NA	NA	9/16
Yamagami^39^	2018	1	2	0	2	0	1	2	0	2	2	2	2	16/24
Yamamoto^40^	2006	2	2	0	2	0	2	2	0	NA	NA	NA	NA	10/16
Yonetani^41^	2012	2	2	0	2	0	1	2	0	NA	NA	NA	NA	9/16

APAC, Asia‐Pacific; MINORS, Methodological Index for Non‐Randomized Studies; NA, not applicable.

**TABLE 4:** Summary of MINORS Score for North America Studies

** Study**		** For All Nonrandomized Studies (Level III‐IV Evidence)**								** Additional Items for Comparative Studies Only**				
** Author**	** Year Published**	** Clearly Stated Aim**	** Consecutive Patient Inclusion**	** Prospective Data Collection**	** Endpoints Appropriate to Aim**	** Unbiased Assessment of Endpoint**	** Follow‐Up Period Appropriate**	** Loss to Follow‐Up <5%**	** Prospective Calculation of the Study Size**	** Adequate Control Group**	** Contemporary Groups**	** Baseline Equivalence of Groups**	** Adequate Statistical Analysis**	** Total**
Backes^42^	2014	2	2	0	2	0	2	2	0	NA	NA	NA	NA	10/16
Bae^43^	2020	2	2	2	2	0	2	2	0	NA	NA	NA	NA	12/16
Baghdadi^7^	2022	2	2	0	2	0	2	2	0	NA	NA	NA	NA	10/16
Boughanem^44^	2011	1	2	0	2	0	2	2	0	NA	NA	NA	NA	9/16
Bram^45^	2025	2	2	0	2	0	1	2	0	NA	NA	NA	NA	9/16
Broughton^46^	2021	2	2	0	2	0	2	2	0	NA	NA	NA	NA	10/16
Byrd^47^	2002	2	2	0	2	0	2	2	0	NA	NA	NA	NA	10/16
Donaldson^48^	2008	1	2	0	2	0	2	0	0	NA	NA	NA	NA	7/16
Edmonds^49^	2010	2	2	0	2	0	2	2	0	NA	NA	NA	NA	10/16
Fabricant^50^	2018	2	2	0	2	1	2	2	0	NA	NA	NA	NA	11/16
Hennrikus^51^	2014	2	2	0	2	1	2	1	0	NA	NA	NA	NA	10/16
Heyworth^52^	2023	NA	NA	NA	NA	NA	NA	NA	NA	NA	NA	NA	NA	NA
Husen^53^	2022	2	2	2	2	0	2	2	0	NA	NA	NA	NA	12/16
Jackson^54^	1989	2	2	0	2	0	2	2	0	NA	NA	NA	NA	10/16
Johnson^55^	2021	2	2	0	2	0	2	2	0	2	2	2	2	16/24
Jones^56^	2010	2	2	0	2	0	2	2	0	NA	NA	NA	NA	10/16
Kocher^57^	2007	2	2	0	2	0	2	0	0	NA	NA	NA	NA	8/16
Kocher^58^	2001	2	2	0	2	2	2	0	0	NA	NA	NA	NA	10/16
Kramer^59^	2015	2	2	0	2	0	2	2	0	NA	NA	NA	NA	10/16
Kramer^60^	2015	2	2	0	2	2	2	1	0	NA	NA	NA	NA	11/16
Lewine^61^	2016	2	2	0	2	0	2	2	0	NA	NA	NA	NA	10/16
Obey^62^	2021	2	2	0	2	0	2	1	0	NA	NA	NA	NA	9/16
Pan^63^	2025	2	2	0	2	0	2	2	0	NA	NA	NA	NA	10/16
Ruch^64^	1998	2	2	0	2	0	2	2	0	NA	NA	NA	NA	10/16
Shi^65^	2012	2	2	0	2	0	2	2	0	NA	NA	NA	NA	10/16
Tabaddor^66^	2010	2	2	0	2	0	2	1	0	NA	NA	NA	NA	9/16
Tan^67^	2022	2	2	0	2	0	1	0	0	NA	NA	NA	NA	7/16
Tis^68^	2012	1	2	0	2	0	2	2	0	NA	NA	NA	NA	9/16
Tisano^69^	2022	2	2	0	2	2	1	1	0	NA	NA	NA	NA	10/16
Tivnon^70^	1976	1	2	0	2	0	2	2	0	NA	NA	NA	NA	9/16
Wall^71^	2014	2	2	0	2	0	2	0	0	2	2	2	0	14/24
Wang^72^	2019	2	2	0	2	0	1	2	0	2	2	2	0	15/24
Webb^73^	2013	2	2	0	2	0	2	1	0	NA	NA	NA	NA	9/16
Wulf^74^	2012	2	2	0	2	0	1	2	0	NA	NA	NA	NA	9/16
Yehyawi^75^	2024	2	2	0	2	0	2	0	0	NA	NA	NA	NA	8/16
Zheng^76^	2024	2	2	2	2	0	2	2	0	2	2	2	2	20/24

MINORS, Methodological Index for Non‐Randomized Studies; NA, Not applicable.

## DISCUSSION

The principal finding of this systematic review is the presence of clear regional variations in both epidemiological and treatment strategies for pediatric OCD. Analysis of the APAC studies revealed an overwhelmingly male patient population (680 out of 738, 91.9% men) with OCD lesions predominantly found in the capitellum (23 out of 29, 79.3%). In contrast, studies from NA reported a more balanced sex distribution (713 out of 1290, 55.3% men) and a higher proportion of knee OCDs (13 out of 36, 36.1% in NA compared with 6 out of 29, 20.7% in APAC).

These findings are consistent with existing global epidemiological data on OCD. Traditionally, men were believed to be affected approximately twice as often as women;^78^ however, recent studies have suggested that OCD is particularly prevalent among young, high‐level athletes, which can skew the male‐to‐female ratio—especially in sports and activities more popular among women.^79^ This review supports this trend and aligns with region‐specific literature. For instance, the incidence of capitellar OCD lesions among young male baseball players in Japan is remarkably greater compared with other regions such as Europe and America, with studies reporting 1.8% cumulative incidence in youth baseball cohorts^80^ and up to 3.4% prevalence in other surveys.^81^ Given that 28 of the 29 (96.6%) APAC studies included in this systematic review originated from Japan, this would explain the overwhelming male bias in the findings.

Furthermore, baseball has been Japan's most popular sport since the late 1800s and plays a role in youth athletic culture. Prestigious high school tournaments such as the National High School Baseball Championship in Japan attract significant interest and engagement nationwide. The popularity of this culturally significant overhead throwing sport could potentially explain the predominance of capitellar OCD in APAC studies, instead of the knee.^79^, ^82^

In terms of preoperative imaging, although MRI remains the gold standard for diagnosing and monitoring OCD because of its superior sensitivity and specificity,^83^ plain radiographs are commonly employed as an initial screening tool for OCD because of their accessibility and cost‐effectiveness. This is reflected in both the APAC and NA regions where the most common imaging modality used was radiographs in 750 out of 1344 (55.8%) and 1014 out of 2045 (49.6%), respectively. However, the 2 regions varied significantly in terms of the use of MRI and computed tomography scans. This difference might either suggest a variation in initial diagnostic approaches or could reflect a greater representation of studies from specialized centers in the included NA literature. MRI may be expedited for suspected OCD, particularly early‐stage lesions, to confirm the diagnosis and assess lesion stability. Furthermore, current literature from the NA region advocates the routine use of MRI for early detection and staging.^83^

This systematic review identified significant regional differences in treatment patterns for pediatric OCD. NA studies employed a higher proportion of arthroscopic techniques including debridement, microfracturing, or drilling (1029 out of 1400 [73.5%] compared with 332 out of 801 [41.4%] in APAC), whereas APAC studies showed significantly greater use of osteochondral grafting techniques with autografts or allografts (334 out of 801 [41.7%] compared with 164 out of 1400 [11.7%] in NA). Current literature similarly describes a predominance of arthroscopic interventions in the NA region before osteochondral grafts gained traction in recent years.^84^ In contrast, Japanese and other APAC literature frequently report fragment fixation, bone‐peg grafting, and osteochondral transplantation options in the treatment of OCD.^85^ Differences in OCD lesion stage at presentation, surgeon training and local practice patterns, availability and regulatory status of graft materials, insurance approval pathways, and patient expectations are all possible contributory factors to the observed therapeutic variation in the 2 regions.

Currently, there are limited data specifically comparing the epidemiology, diagnostic approaches, and surgical interventions for pediatric OCD between APAC and NA. Including data from over 2000 patients, this study constitutes one of the largest comparative analyses of pediatric OCD to date.

### Limitations

This study has some limitations that should be acknowledged. First, this analysis relied on study‐level rather than patient‐level data, which precludes adjustment for individual confounders such as sport participation, skeletal maturity, or lesion stage and stability. Considerable heterogeneity was present across the included studies, which ranged from retrospective case to higher‐level cohort studies and a randomized controlled trial. Additionally, variability in reporting standards across studies also posed challenges. For instance, multiple studies failed to report important details such as patient's age, sex, and follow‐up duration. Finally, the level of evidence of this review is limited by the lack of appropriately powered RCTs in the literature, thus precluding direct comparisons between the variables studied and further pooling of data outcomes through meta‐analysis. The inclusion of lower‐level study designs such as retrospective studies also meant that this study was susceptible to the biases inherent to that study design.

## CONCLUSIONS

There are significant regional variations in the epidemiology, imaging modalities, and surgical management for pediatric OCD. The predominantly male bias in the APAC region and greater use of MRI and arthroscopic techniques in the NA region reflect underlying biological, cultural, and health care‐related differences.

## DISCLOSURES

The authors (J.R.Y.W., Y.X.L., S.H.S.T., J.H.P.H., M.W.) declare that they have no known competing financial interests or personal relationships that could have appeared to influence the work reported in this article.

## References

[1] Edmonds EW , Shea KG . Osteochondritis dissecans: Editorial comment. Clin Orthop Relat Res. 2013;471:1105‐1106. 10.1007/s11999-013-2837-6 PMC3586031 23404419

[2] Kessler JI , Nikizad H , Shea KG , Jacobs JC Jr , Bebchuk JD , Weiss JM . The demographics and epidemiology of osteochondritis dissecans of the knee in children and adolescents. Am J Sports Med. 2014;42:320‐326. 10.1177/0363546513510390 24272456

[3] Kessler JI , Weiss JM , Nikizad H , et al. Osteochondritis dissecans of the ankle in children and adolescents: Demographics and epidemiology. Am J Sports Med. 2014;42:2165‐2171. 10.1177/0363546514538406 24989493

[4] Kessler JI , Jacobs JC Jr , Cannamela PC , Weiss JM , Shea KG . Demographics and epidemiology of osteochondritis dissecans of the elbow among children and adolescents. Orthop J Sports Med. 2018;6:2325967118815846. 10.1177/2325967118815846 PMC6302285 30622996

[5] Wall E , Von Stein D . Juvenile osteochondritis dissecans. Orthop Clin North Am. 2003;34:341‐353. 10.1016/s0030-5898(03)00038-5 12974484

[6] Sharma DK , Kumar N , Lal H , Sahu BK , Saikia SS . Osteochondritis dissecans—Does platelet rich plasma really help. J Clin Orthop Trauma. 2018;9:153‐156. 10.1016/j.jcot.2017.09.020 PMC5995159 29896019

[7] Baghdadi S , Isaacs D , Chan CT , Wells L , Ganley TJ , Lawrence JTR . Arthroscopic drilling for stable juvenile osteochondritis dissecans of the knee is safe and patients reliably return to daily activities by 3 months. Arthrosc Sports Med Rehabil. 2022;4:e645‐e651. 10.1016/j.asmr.2021.12.006 PMC9042903 35494308

[8] Sadr KN , Pulido PA , McCauley JC , Bugbee WD . Osteochondral allograft transplantation in patients with osteochondritis dissecans of the knee. Am J Sports Med. 2016;44:2870‐2875. 10.1177/0363546516657526 27496906

[9] Quigley R , Allahabadi S , Yazdi AA , et al. Bioabsorbable screw fixation provides good results with low failure rates at mid‐term follow‐up of stable osteochondritis dissecans lesions that do not improve with initial conservative treatment. Arthrosc Sports Med Rehabil. 2024;6:100863. 10.1016/j.asmr.2023.100863 PMC10867764 38362480

[10] Chambers HG , Shea KG , Anderson AF , et al. Diagnosis and treatment of osteochondritis dissecans. J Am Acad Orthop Surg. 2011;19:297‐306. 10.5435/00124635-201105000-00007 21536629

[11] Moher D , Liberati A , Tetzlaff J , Altman DG , PRISMA Group . Preferred reporting items for systematic reviews and meta‐analyses: The PRISMA statement. PLoS Med. 2009;6:e1000097. PMC3090117 21603045

[12] Hozo SP , Djulbegovic B , Hozo I . Estimating the mean and variance from the median, range, and the size of a sample. BMC Med Res Methodol. 2005;5:13. 10.1186/1471-2288-5-13 PMC1097734 15840177

[13] Adachi N , Deie M , Nakamae A , Ishikawa M , Motoyama M , Ochi M . Functional and radiographic outcome of stable juvenile osteochondritis dissecans of the knee treated with retroarticular drilling without bone grafting. Arthroscopy. 2009;25:145‐152. 10.1016/j.arthro.2008.09.008 19171273

[14] Adachi N , Deie M , Nakamae A , Okuhara A , Kamei G , Ochi M . Functional and radiographic outcomes of unstable juvenile osteochondritis dissecans of the knee treated with lesion fixation using bioabsorbable pins. J Pediatr Orthop. 2015;35:82‐88. 10.1097/BPO.0000000000000226 24919133

[15] Arai Y , Hara K , Inoue H , et al. Early clinical results of arthroscopically assisted drilling via the radius in a distal‐to‐proximal direction for osteochondritis dissecans of the elbow. Orthop J Sports Med. 2019;7:2325967119868937. 10.1177/2325967119868937 PMC6732861 31523694

[16] Chen H , Xu W , Hu N , Jiang D , Huang W . Arthroscopic antegrade drilling for unstable juvenile osteochondritis dissecans of the knee: Mid‐term results. Arch Orthop Trauma Surg. 2015;135:1727‐1732. 10.1007/s00402-015-2332-5 26385562

[17] Funakoshi T , Momma D , Matsui Y , et al. Autologous osteochondral mosaicplasty for centrally and laterally located, advanced capitellar osteochondritis dissecans in teenage athletes: Clinical outcomes, radiography, and magnetic resonance imaging findings. Am J Sports Med. 2018;46:1943‐1951. 10.1177/0363546518768279 29738680

[18] Imada H , Mori R , Shibuya H , et al. Lateral wall fixation with bone pegs for advanced osteochondritis dissecans of the humeral capitellum. JSES Int. 2020;5:35‐41. 10.1016/j.jseint.2020.09.002 PMC7846702 33554161

[19] Iwasaki N , Kato H , Ishikawa J , Saitoh S , Minami A . Autologous osteochondral mosaicplasty for capitellar osteochondritis dissecans in teenaged patients. Am J Sports Med. 2006;34:1233‐1239. 10.1177/0363546506286343 16567456

[20] Iwasaki N , Kato H , Kamishima T , Minami A . Sequential alterations in magnetic resonance imaging findings after autologous osteochondral mosaicplasty for young athletes with osteochondritis dissecans of the humeral capitellum. Am J Sports Med. 2009;37:2349‐2354. 10.1177/0363546509341793 19726624

[21] Kawasaki K , Uchio Y , Adachi N , Iwasa J , Ochi M . Drilling from the intercondylar area for treatment of osteochondritis dissecans of the knee joint. Knee. 2003;10:257‐263. 10.1016/s0968-0160(02)00104-7 12893148

[22] Kiyomatsu H , Takeba J , Imai H , et al. Treatment of osteochondritis dissecans of the humeral capitellum with a fragment fixation method using absorbable pins. JSES Int. 2021;5:564‐570. 10.1016/j.jseint.2020.12.017 PMC8178622 34136872

[23] Kosaka M , Nakase J , Takahashi R , et al. Outcomes and failure factors in surgical treatment for osteochondritis dissecans of the capitellum. J Pediatr Orthop. 2013;33:719‐724. 10.1097/BPO.0b013e3182924662 23774205

[24] Maruyama M , Harada M , Satake H , Tomohiro U , Takagi M , Takahara M . Bone‐peg grafting for osteochondritis dissecans of the humeral capitellum. J Orthop Surg (Hong Kong). 2016;24:51‐56. 10.1177/230949901602400113 27122513

[25] Maruyama M , Takahara M , Harada M , Satake H , Takagi M . Outcomes of an open autologous osteochondral plug graft for capitellar osteochondritis dissecans: Time to return to sports. Am J Sports Med. 2014;42:2122‐2127. 10.1177/0363546514538759 24950681

[26] Matsuura T , Hashimoto Y , Nishino K , Nishida Y , Takahashi S , Shimada N . Comparison of clinical and radiographic outcomes between central and lateral lesions after osteochondral autograft transplantation for osteochondritis dissecans of the humeral capitellum. Am J Sports Med. 2017;45:3331‐3339. 10.1177/0363546517730358 28968135

[27] Matsuura T , Iwame T , Suzue N , et al. Long‐term outcomes of arthroscopic debridement with or without drilling for osteochondritis dissecans of the capitellum in adolescent baseball players: A ≥10‐year follow‐up study. Arthroscopy. 2020;36:1273‐1280. 10.1016/j.arthro.2020.01.020 32001276

[28] Mihara K , Suzuki K , Makiuchi D , Nishinaka N , Yamaguchi K , Tsutsui H . Surgical treatment for osteochondritis dissecans of the humeral capitellum. J Shoulder Elbow Surg. 2010;19:31‐37. 10.1016/j.jse.2009.04.007 19556146

[29] Miyake J , Masatomi T . Arthroscopic debridement of the humeral capitellum for osteochondritis dissecans: Radiographic and clinical outcomes. J Hand Surg Am. 2011;36:1333‐1338. 10.1016/j.jhsa.2011.05.024 21705155

[30] Nishimura A , Morita A , Fukuda A , Kato K , Sudo A . Functional recovery of the donor knee after autologous osteochondral transplantation for capitellar osteochondritis dissecans. Am J Sports Med. 2011;39:838‐842. 10.1177/0363546510388386 21189356

[31] Nishinaka N , Tsutsui H , Yamaguchi K , Uehara T , Nagai S , Atsumi T . Costal osteochondral autograft for reconstruction of advanced‐stage osteochondritis dissecans of the capitellum. J Shoulder Elbow Surg. 2014;23:1888‐1897. 10.1016/j.jse.2014.06.047 25240513

[32] Oshiba H , Itsubo T , Ikegami S , Nakamura K , Uchiyama S , Kato H . Results of bone peg grafting for capitellar osteochondritis dissecans in adolescent baseball players. Am J Sports Med. 2016;44:3171‐3178. 10.1177/0363546516658038 27514737

[33] Shimada K , Temporin K , Oura K , Tanaka H , Noguchi R . Anconeus muscle‐pedicle bone graft with periosteal coverage for osteochondritis dissecans of the humeral capitellum. Orthop J Sports Med. 2017;5:2325967117727531. 10.1177/2325967117727531 PMC5602222 28944252

[34] Takeba J , Takahashi T , Watanabe S , et al. Short‐term clinical results of arthroscopic osteochondral fixation for elbow osteochondritis dissecans in teenaged baseball players. J Shoulder Elbow Surg. 2015;24:1749‐1756. 10.1016/j.jse.2015.07.009 26480880

[35] Uchida S , Utsunomiya H , Taketa T , et al. Arthroscopic fragment fixation using hydroxyapatite/poly‐L‐lactate acid thread pins for treating elbow osteochondritis dissecans. Am J Sports Med. 2015;43:1057‐1065. 10.1177/0363546515570871 25737319

[36] Ueda Y , Sugaya H , Takahashi N , et al. Arthroscopic fragment resection for capitellar osteochondritis dissecans in adolescent athletes: 5‐ to 12‐year follow‐up. Orthop J Sports Med. 2017;5:2325967117744537. 10.1177/2325967117744537 PMC5734470 29276715

[37] Ueda Y , Sugaya H , Takahashi N , et al. Comparison between osteochondral autograft transplantation and arthroscopic fragment resection for large capitellar osteochondritis dissecans in adolescent athletes: A minimum 5 years’ follow‐up. Am J Sports Med. 2021;49:1145‐1151. 10.1177/0363546521994558 33705216

[38] Ueki M , Moriya K , Yoshizu T , Tsubokawa N , Kouda H , Endo N . Closed‐wedge osteotomy of the distal humerus for treating osteochondritis dissecans of the capitellum in young patients. Orthop J Sports Med. 2019;7:2325967119876247. 10.1177/2325967119876247 PMC6820184 31696130

[39] Yamagami N , Yamamoto S , Aoki A , Ito S , Uchio Y . Outcomes of surgical treatment for osteochondritis dissecans of the elbow: Evaluation by lesion location. J Shoulder Elbow Surg. 2018;27:2262‐2270. 10.1016/j.jse.2018.08.011 30446232

[40] Yamamoto Y , Ishibashi Y , Tsuda E , Sato H , Toh S . Osteochondral autograft transplantation for osteochondritis dissecans of the elbow in juvenile baseball players: Minimum 2‐year follow‐up. Am J Sports Med. 2006;34:714‐720. 10.1177/0363546505282620 16382006

[41] Yonetani Y , Tanaka Y , Shiozaki Y , et al. Transarticular drilling for stable juvenile osteochondritis dissecans of the medial femoral condyle. Knee Surg Sports Traumatol Arthrosc. 2012;20:1528‐1532. 10.1007/s00167-011-1736-1 22072323

[42] Backes JR , Durbin TC , Bentley JC , Klingele KE . Multifocal juvenile osteochondritis dissecans of the knee: A case series. J Pediatr Orthop. 2014;34:453‐458. 10.1097/BPO.0000000000000121 24248591

[43] Bae DS , Ingall EM , Miller PE , Eisenberg K . Early results of single‐plug autologous osteochondral grafting for osteochondritis dissecans of the capitellum in adolescents. J Pediatr Orthop. 2020;40:78‐85. 10.1097/BPO.0000000000001114 31923167

[44] Boughanem J , Riaz R , Patel RM , Sarwark JF . Functional and radiographic outcomes of juvenile osteochondritis dissecans of the knee treated with extra‐articular retrograde drilling. Am J Sports Med. 2011;39:2212‐2217. 10.1177/0363546511416594 21828362

[45] Bram JT , Jones RH , Cogsil T , Beber SA , Gross PW , Fabricant PD . Outcomes of grafting extracellular matrix with bone marrow aspirate concentrate in pediatric and adolescent patients with capitellar osteochondritis dissecans. J Shoulder Elbow Surg. 2025;34:2015‐2021. 10.1016/j.jse.2024.12.008 39863157

[46] Broughton JS , Goldfarb CA , Obey MR , Smith MV . Performance of patient‐reported outcomes measurement information system (PROMIS) scores compared with legacy metrics in evaluating outcomes after surgical treatment for osteochondritis dissecans of the humeral capitellum. J Shoulder Elbow Surg. 2021;30:1511‐1518. 10.1016/j.jse.2020.11.032 33486062

[47] Byrd JW , Jones KS . Arthroscopic surgery for isolated capitellar osteochondritis dissecans in adolescent baseball players: Minimum three‐year follow‐up. Am J Sports Med. 2002;30:474‐478. 10.1177/03635465020300040401 12130399

[48] Donaldson LD , Wojtys EM . Extraarticular drilling for stable osteochondritis dissecans in the skeletally immature knee. J Pediatr Orthop. 2008;28:831‐835. 10.1097/BPO.0b013e31818ee248 19034173

[49] Edmonds EW , Albright J , Bastrom T , Chambers HG . Outcomes of extra‐articular, intra‐epiphyseal drilling for osteochondritis dissecans of the knee. J Pediatr Orthop. 2010;30:870‐878. 10.1097/BPO.0b013e3181f5a216 21102215

[50] Fabricant PD , Yen YM , Kramer DE , et al. Fixation of traumatic chondral‐only fragments of the knee in pediatric and adolescent athletes: A retrospective multicenter report. Orthop J Sports Med. 2018;6:2325967117753140. 10.1177/2325967117753140 PMC5808975 29450207

[51] Hennrikus WP , Miller PE , Micheli LJ , Waters PM , Bae DS . Internal fixation of unstable in situ osteochondritis dissecans lesions of the capitellum. J Pediatr Orthop. 2015;35:467‐473. 10.1097/BPO.0000000000000308 25264554

[52] Heyworth BE , Ganley TJ , Liotta ES , et al. Transarticular versus retroarticular drilling of stable osteochondritis dissecans of the knee: A prospective multicenter randomized controlled trial by the ROCK group. Am J Sports Med. 2023;51:1392‐1402. 10.1177/03635465231165290 37039536

[53] Husen M , Krych AJ , Stuart MJ , Milbrandt TA , Saris DBF . Successful fixation of traumatic articular cartilage‐only fragments in the juvenile and adolescent knee: A case series. Orthop J Sports Med. 2022;10:23259671221138074. 10.1177/23259671221138074 PMC9706060 36458110

[54] Jackson DW , Silvino N , Reiman P . Osteochondritis in the female gymnast's elbow. Arthroscopy. 1989;5:129‐136. 10.1016/0749-8063(89)90008-x 2736009

[55] Johnson MA , Park K , Talwar D , Maguire KJ , Lawrence JTR . Predicting outcomes of talar osteochondritis dissecans lesions in children. Orthop J Sports Med. 2021;9:23259671211051769. 10.1177/23259671211051769 PMC8597075 34805420

[56] Jones KJ , Wiesel BB , Sankar WN , Ganley TJ . Arthroscopic management of osteochondritis dissecans of the capitellum: Mid‐term results in adolescent athletes. J Pediatr Orthop. 2010;30:8‐13. 10.1097/BPO.0b013e3181c3be83 20032735

[57] Kocher MS , Czarnecki JJ , Andersen JS , Micheli LJ . Internal fixation of juvenile osteochondritis dissecans lesions of the knee. Am J Sports Med. 2007;35:712‐718. 10.1177/0363546506296608 17337729

[58] Kocher MS , Micheli LJ , Yaniv M , Zurakowski D , Ames A , Adrignolo AA . Functional and radiographic outcome of juvenile osteochondritis dissecans of the knee treated with transarticular arthroscopic drilling. Am J Sports Med. 2001;29:562‐566. 10.1177/03635465010290050701 11573913

[59] Kramer DE , Glotzbecker MP , Shore BJ , et al. Results of surgical management of osteochondritis dissecans of the ankle in the pediatric and adolescent population. J Pediatr Orthop. 2015;35:725‐733. 10.1097/BPO.0000000000000352 25393568

[60] Kramer DE , Yen YM , Simoni MK , et al. Surgical management of osteochondritis dissecans lesions of the patella and trochlea in the pediatric and adolescent population. Am J Sports Med. 2015;43:654‐662. 10.1177/0363546514562174 25556222

[61] Lewine EB , Miller PE , Micheli LJ , Waters PM , Bae DS . Early results of drilling and/or microfracture for grade IV osteochondritis dissecans of the capitellum. J Pediatr Orthop. 2016;36:803‐809. 10.1097/BPO.0000000000000575 26090983

[62] Obey MR , Goldfarb CA , Broughton JS , Gebhart SS , Smith MV . Early improvement in patient‐reported outcome scores after operative treatment of osteochondritis dissecans of the humeral capitellum. J Hand Surg Am. 2021;46:1120.e1‐1120.e7. 10.1016/j.jhsa.2021.03.003 33931273

[63] Pan T , Olson SN , Giuffrida BM , Pinamont W , Hennrikus WL . Osteochondritis dissecans lesions of the talus in adolescent athletes: Outcomes and management of arthroscopic surgery. J Orthop Rep. 2025;4:100478.

[64] Ruch DS , Cory JW , Poehling GG . The arthroscopic management of osteochondritis dissecans of the adolescent elbow. Arthroscopy. 1998;14:797‐803. 10.1016/s0749-8063(98)70013-1 9848588

[65] Shi LL , Bae DS , Kocher MS , Micheli LJ , Waters PM . Contained versus uncontained lesions in juvenile elbow osteochondritis dissecans. J Pediatr Orthop. 2012;32:221‐225. 10.1097/BPO.0b013e31824afecf 22411324

[66] Tabaddor RR , Banffy MB , Andersen JS , et al. Fixation of juvenile osteochondritis dissecans lesions of the knee using poly 96L/4D‐lactide copolymer bioabsorbable implants. J Pediatr Orthop. 2010;30:14‐20. 10.1097/BPO.0b013e3181c6318c 20032736

[67] Tan XW , Joukhadar N , Leduc S , Aubin CÉ , Hupin M , Nault ML . Outcome of retroarticular drilling for osteochondritis dissecans of the talus in a pediatric population. Foot Ankle Surg. 2022;28:628‐634. 10.1016/j.fas.2021.07.005 34330660

[68] Tis JE , Edmonds EW , Bastrom T , Chambers HG . Short‐term results of arthroscopic treatment of osteochondritis dissecans in skeletally immature patients. J Pediatr Orthop. 2012;32:226‐231. 10.1097/BPO.0b013e31824afeb8 22411325

[69] Tisano B , Ellis HB , Wyatt C , Wilson PL . Osteochondral allograft for unsalvageable osteochondritis dissecans in the skeletally immature knee. Orthop J Sports Med. 2022;10:23259671211072515. 10.1177/23259671211072515 PMC8844736 35178463

[70] Tivnon MC , Anzel SH , Waugh TR . Surgical management of osteochondritis dissecans of the capitellum. Am J Sports Med. 1976;4:121‐128. 10.1177/036354657600400305 984288

[71] Wall EJ , Heyworth BE , Shea KG , et al. Trochlear groove osteochondritis dissecans of the knee patellofemoral joint. J Pediatr Orthop. 2014;34:625‐630. 10.1097/BPO.0000000000000212 25126843

[72] Wang KK , Bixby SD , Bae DS . Osteochondritis dissecans of the humeral trochlea: Characterization of a rare disorder based on 28 cases. Am J Sports Med. 2019;47:2167‐2173. 10.1177/0363546519855036 31233330

[73] Webb JE , Lewallen LW , Christophersen C , Krych AJ , McIntosh AL . Clinical outcome of internal fixation of unstable juvenile osteochondritis dissecans lesions of the knee. Orthopedics. 2013;36:e1444‐e1449. 10.3928/01477447-20131021-30 24200451

[74] Wulf CA , Stone RM , Giveans MR , Lervick GN . Magnetic resonance imaging after arthroscopic microfracture of capitellar osteochondritis dissecans. Am J Sports Med. 2012;40:2549‐2556. 10.1177/0363546512458765 23019252

[75] Yehyawi S , Zielinski EM , Bartkiw M , Peck K , Hastings H 2nd. Five‐year follow‐up of adolescent gymnasts after surgical treatment of osteochondritis dissecans of the elbow. J Hand Surg Am. 2024;49:934.e1‐934.e6. 10.1016/j.jhsa.2022.12.009 37115144

[76] Zheng ET , Osada K , Williams KA , Bae DS . Treatment and early outcomes of capitellar osteochondritis dissecans. Am J Sports Med. 2024;52:3466‐3472. 10.1177/03635465241289939 39547213

[77] Slim K , Nini E , Forestier D , Kwiatkowski F , Panis Y , Chipponi J . Methodological index for non‐randomized studies (minors): Development and validation of a new instrument. ANZ J Surg. 2003;73:712‐716. 10.1046/j.1445-2197.2003.02748.x 12956787

[78] Dexel M , Doerig M . 8. Osteochondrosis dissecans, 10‐ und Mehrjahres‐Ergebnisse. a). Spätresultate nach konservativer und operativer Behandlung der Osteochondrosis dissecans am Kniegelenk. Orthopade. 1979;8:120‐126 [in German]. 542275

[79] Chau MM , Klimstra MA , Wise KL , et al. Osteochondritis dissecans: Current understanding of epidemiology, etiology, management, and outcomes. J Bone Joint Surg Am. 2021;103:1132‐1151. 10.2106/JBJS.20.01399 PMC8272630 34109940

[80] Matsuura T , Iwame T , Suzue N , et al. Cumulative incidence of osteochondritis dissecans of the capitellum in preadolescent baseball players. Arthroscopy. 2019;35:60‐66. 10.1016/j.arthro.2018.08.034 30611367

[81] Kida Y , Morihara T , Kotoura Y , et al. Prevalence and clinical characteristics of osteochondritis dissecans of the humeral capitellum among adolescent baseball players. Am J Sports Med. 2014;42:1963‐1971. 10.1177/0363546514536843 24944293

[82] Wood D , Davis DD , Carter KR . Osteochondritis dissecans. In: StatPearls. Treasure Island (FL): StatPearls Publishing; 2023. 30252347

[83] Quatman CE , Quatman‐Yates CC , Schmitt LC , Paterno MV . The clinical utility and diagnostic performance of MRI for identification and classification of knee osteochondritis dissecans. J Bone Joint Surg Am. 2012;94:1036‐1044. 10.2106/JBJS.K.00275 PMC3364031 22637210

[84] Bram J , Gross P , Fabricant P . Osteochondral autograft transplantation for OCD: Techniques and outcomes. Oper Techn Sports Med. 2023;31:151006.

[85] Sato K , Iwamoto T , Matsumura N , et al. Costal osteochondral autograft for advanced osteochondritis dissecans of the humeral capitellum in adolescent and young adult athletes: Clinical outcomes with a mean follow‐up of 4.8 years. J Bone Joint Surg Am. 2018;100:903‐913. 10.2106/JBJS.17.01035 29870440

